# Environmental predictors of West Nile fever risk in Europe

**DOI:** 10.1186/1476-072X-13-26

**Published:** 2014-07-01

**Authors:** Annelise Tran, Bertrand Sudre, Shlomit Paz, Massimiliano Rossi, Annie Desbrosse, Véronique Chevalier, Jan C Semenza

**Affiliations:** 1 CIRAD, UPR Animal et Gestion Intégrée des Risques, Montpellier, France; 2 CIRAD, UMR Territoires Environnement Télédétection et Information Spatiale, Montpellier, France; 3 Surveillance and Response Support, European Centre for Disease Prevention and Control, Surveillance and Response Support, Stockholm, Sweden; 4 Department of Geography and Environmental Studies, University of Haifa, Mt. Carmel, Haifa, Israel; 5 Head of Health Determinants Programme, Office of the Chief Scientist, European Centre for Disease Prevention and Control, Office of the Chief Scientist, Stockholm, SE-171 83, Sweden

**Keywords:** West nile fever, West nile virus, Environmental determinants, Epidemiology, Temperature, Surveillance, Arbovirus, Remote sensing, Risk maps

## Abstract

**Background:**

West Nile virus (WNV) is a mosquito-borne pathogen of global public health importance. Transmission of WNV is determined by abiotic and biotic factors. The objective of this study was to examine environmental variables as predictors of WNV risk in Europe and neighboring countries, considering the anomalies of remotely sensed water and vegetation indices and of temperature at the locations of West Nile fever (WNF) outbreaks reported in humans between 2002 and 2013.

**Methods:**

The status of infection by WNV in relationship to environmental and climatic risk factors was analyzed at the district level using logistic regression models. Temperature, remotely sensed Normalized Difference Vegetation Index (NDVI) and Modified Normalized Difference Water Index (MNDWI) anomalies, as well as population, birds’ migratory routes, and presence of wetlands were considered as explanatory variables.

**Results:**

The anomalies of temperature in July, of MNDWI in early June, the presence of wetlands, the location under migratory routes, and the occurrence of a WNF outbreak the previous year were identified as risk factors. The best statistical model according to the Akaike Information Criterion was used to map WNF risk areas in 2012 and 2013. Model validations showed a good level of prediction: area under Receiver Operator Characteristic curve = 0.854 (95% Confidence Interval 0.850-0.856) for internal validation and 0.819 (95% Confidence Interval 0.814-0.823) (2012) and 0.853 (95% Confidence Interval 0.850-0.855) (2013) for external validations, respectively.

**Conclusions:**

WNF incidence is increasing in Europe and WNV is expanding into new areas where it had never been observed before. Our model can be used to direct surveillance activities and public health interventions for the upcoming WNF season.

## Background

West Nile virus (WNV), a member of the *Flavivirus* genus (family *Flaviviridae),* is responsible for West Nile disease (WND), which causes considerable morbidity and mortality worldwide. WND, including West Nile fever (WNF) and West Nile neuro invasive disease (WNND), is an emerging arbovirosis originating from the Old World which was introduced to the Americas in 1999 [1]. Today WNV is the most widespread flavivirus in the world, with a geographic distribution covering Africa, Eurasia, and the Americas. The public health burden with the associated human and economic costs present a strong case for antecedent estimates of WND risk. Wild birds are the principal hosts and critical to support enzootic cycles of WNV and virus dispersal [2]. WNV is transmitted from birds to birds by ornithophilic mosquitoes, predominantly of the *Culex* genus [3]. WNV is pathogenic for horses and humans who are accidental hosts [4].

The transmission, epidemiology and geographic distribution of WND is a complex function of biotic (*i.e.* host abundance and diversity, bird migration, mosquito distribution, etc.) and abiotic (*i.e.* physical features of the environment and climate) factors. Both biological factors (*i.e.* vectors diversity, survival of pathogens within the vectors) and environmental determinants (*i.e.* climatic conditions, availability of water bodies) have impacts on the transmission cycles [2,5].

WN virus circulation has been confirmed at erratic intervals in the last decades in several countries of the Mediterranean basin (*e.g.*[6-9]) and in Eastern and Central Europe (*e.g.*[10]). However, in the summer of 2010, the number of WND cases in previously uninfected areas in Europe and its neighboring countries was the highest ever reported [11]. In a recent study, positive temperature anomalies were identified as major risk factors in WND outbreak occurrence in 2010 in Europe [12], but environmental variables derived from Earth Observation data had not been tested yet in the European context over large areas [13].

Thus, in this study we examined meteorological and environmental variables related to the temperature, the state of vegetation and water bodies, and birds’ migratory routes as predictors of WND risk.

## Results and discussion

### WND outbreaks, 2002–2013

**Figure 1:**
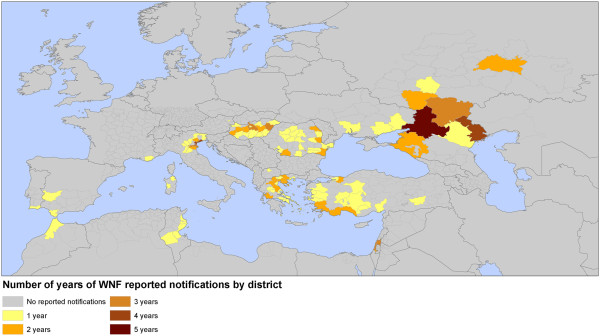
**Map of West Nile fever outbreaks by year, Europe and neighboring countries between 2002 and 2011.** Note: The map represents the number of WNF presence notification by year over the study period (2002–2011).

Between 2002 and 2009 WND outbreaks in humans were confined to a restricted number of districts in Europe and neighboring countries (Table 1). Sustained transmission within one district over a number of years was rarely observed. However, in the summer of 2010, Europe and its neighboring countries experienced an unprecedented upsurge in the number of WND cases: in Eastern Europe, particularly in Russia, Greece, Romania, and Hungary, and in two Mediterranean countries, Israel and Turkey (Figure 1). In Western Europe, two countries, Spain and Italy, reported a limited number of cases.The outbreaks in Europe and Eurasia during the summers of 2011 and 2012 followed most of the disease locations of 2010 (except in Spain), and reached new areas such as Ukraine, the Western Balkans, Tunisia, and Algeria (Figure 1). As in 2010, most of the cases were reported between the end of July and the end of September. In 2013, 97 districts were affected notably in Italy and in central Europe (Hungary, Romania and Serbia) with persistent circulation of WNV in Russia. Tunisia was also affected.

**Table 1:** West Nile Fever outbreaks, Europe and neighboring countries, 2002–2013

**Country**	**Number of districts reporting WND cases in humans**											
	**2002**	**2003**	**2004**	**2005**	**2006**	**2007**	**2008**	**2009**	**2010**	**2011**	**2012**	**2013**
Albania	0	0	0	0	0	0	0	0	0	1	0	0
Algeria	0	0	0	0	0	0	0	0	0	0	1	0
Bosnia and Herzegovina	0	0	0	0	0	0	0	0	0	0	0	2
Bulgaria	0	0	0	0	0	0	0	0	0	0	1	0
Croatia	0	0	0	0	0	0	0	0	0	0	3	2
France	0	1	0	0	0	0	0	0	0	0	0	0
Greece	0	0	0	0	0	0	0	0	12	11	16	9
Hungary	0	≥1	≥1	≥1	≥1	≥1	9	0	11	0	13	11
Israel	5	≥1	≥1	0	0	0	0	0	5	4	5	4
Italy	0	0	0	0	0	0	4	7	3	7	9	15
Kosovo	0	0	0	0	0	0	0	0	0	0	3	0
Macedonia	0	0	0	0	0	0	0	0	0	1	4	1
Montenegro	0	0	0	0	0	0	0	0	0	0	1	3
Morocco	0	1	0	0	0	0	0	0	0	0	0	0
Occupied Palestinian territory	0	0	0	0	1	0	0	0	0	0	2	0
Portugal	0	0	1	0	0	0	0	0	0	0	0	0
Romania	0	0	0	0	0	0	≥1	1	19	5	6	11
Russia	1	0	0	3	1	1	0	0	7	7	13	12
Serbia	0	0	0	0	0	0	0	0	0	0	5	18
Spain	0	0	1	0	0	0	0	0	1	0	0	0
Tunisia	0	5	0	0	0	0	0	0	0	1	9	5
***Total***	*6*	*≥9*	*≥4*	*≥4*	*≥3*	*≥2*	*≥14*	*8*	*73*	*41*	*92*	*94*

### Temperature anomalies

**Figure 2:**
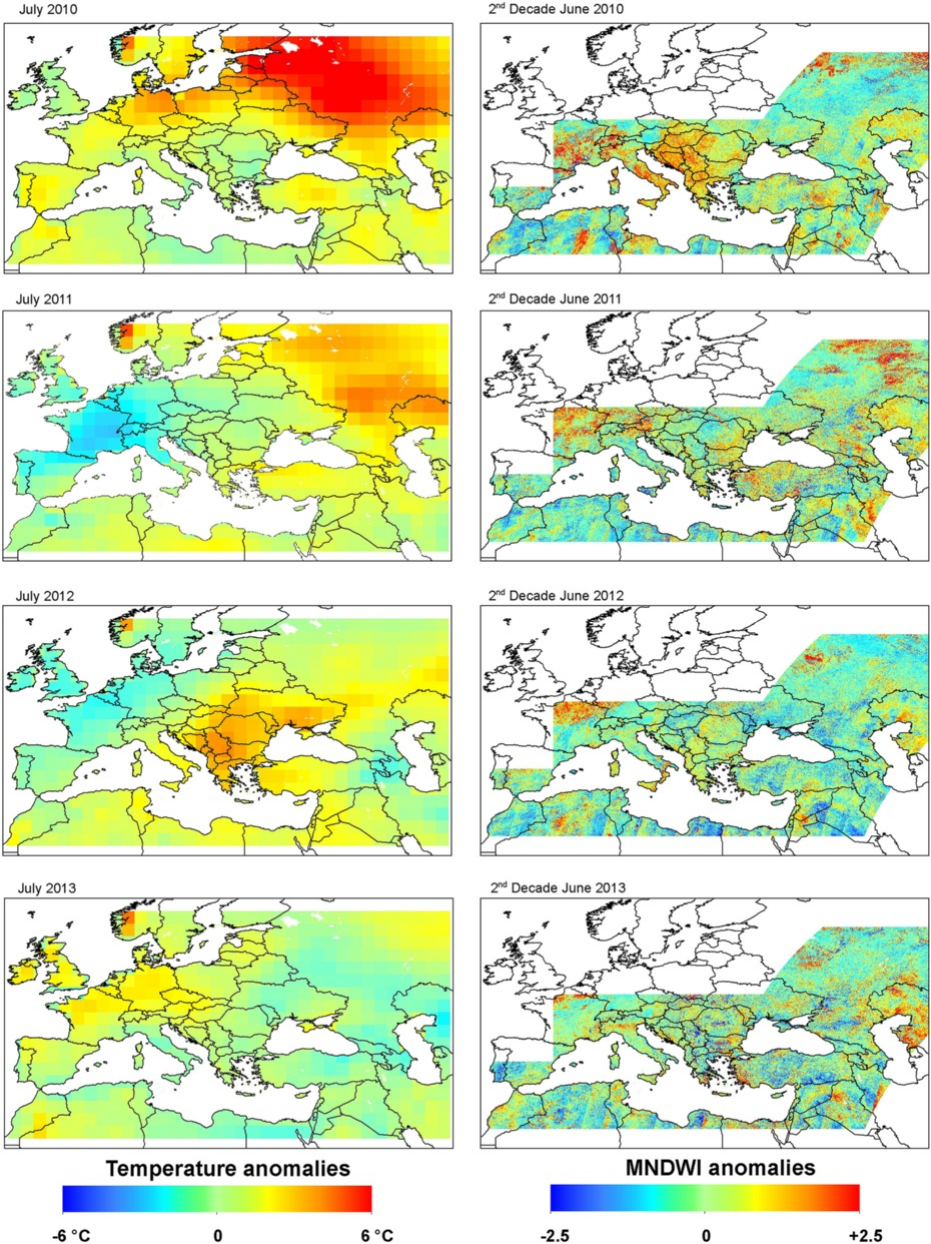
**Anomalies of temperatures and MNDWI values, 2010–2013. a)** Monthly anomalies from the perennial mean monthly temperature (July). **b)** MNDWI anomalies from the 2002–2011 average (21st MODIS period).

Figure 2a presents the monthly anomalies of temperatures for selected month (July) for the years 2010–2013. The maps show that while spring 2010 was very hot in North Africa, the summer of 2010 was severely warm in Eurasia, with extreme anomalies in July (above 6°C) and August. Spring 2011 was warm above normal in Western Europe and the summer was warm in June and August in the central and southern parts of the continent. Again, Eurasia was very warm in July. In 2012, the temperatures of the hot season were above the perennial averages mainly in Eurasia and Southern Europe.

### Water index anomalies

Figure 2b presents the anomalies of Modified Normalized Difference Water Index (MNDWI) for one selected Moderate Resolution Imaging Spectroradiometer (MODIS) composite 8-days period (21st MODIS 8-day period: 9th – 16th June) for the years 2010–2013. Maps highlight a high heterogeneity in the anomalies of the water index derived from MODIS imagery throughout Europe. Higher anomalies were observed in Southern and Eastern Europe in June 2010.

### Environmental risk factors of WNV infection in Europe

**Figure 3:**
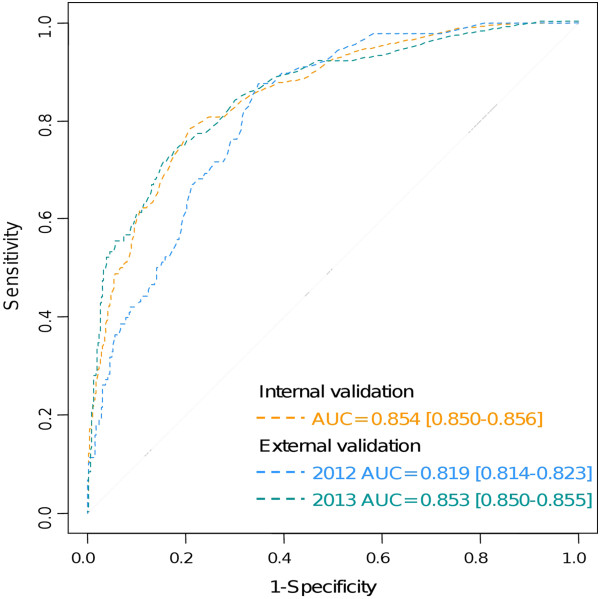
ROC curves of the best model of the probability of WNV infection.

After the univariate analysis, 12 covariates were kept for multi-variate analysis (see Additional file 1: Table S1). According to the Akaike Information Criterion (AIC) values, the probability of infection by WNV at the district level is better explained as a function of the anomalies of temperature in July, of MNDWI in the 21^st^ MODIS-8 day period (i.e. June 9^th^-16^th^), the weighted average of the number of infected districts amongst the neighborhood the previous year (λ), the presence of wetlands, the type of passerine migratory routes, the population, all positively and highly significantly (p < 0.01) correlated with the probability of infection (Table 2). Normalized Difference Vegetation Index (NDVI) variables were not retained as risk factor for WNV infection. Inspection of the residuals from the final model showed no significant spatial autocorrelation (Durbin-Watson test = 1.99, p-value = 0.23).Area Under Curve (AUC) of the model was 0.854 (95% Confidence Interval (CI) 0.850-0.856) for internal validation, 0.819 (95% CI 0.814-0.823) (2012 data) and 0.853 (95% CI 0.850-0.855) (2013 data) for external validation, indicating good predictions (Receiver operator characteristic curve (ROC) curves are presented in Figure 3).

**Table 2:** Multivariate logistic regression model parameter of the risk of WNV infection at district level, EU and neighbouring countries

	**Parameter**	**95% CI**	**p-value**
Intercept	−5.85	[−6.02;-5.74]	-
**TMPJUL**	**0.37**	[0.32;0.41]	**<10**^**−7**^
**MNDWI21**	**1.14**	[1.06;1.22]	**<10**^**−15**^
**λ**	**5.06**	[4.78;5.31]	**<10**^**−15**^
WETLANDS			
Absence			
**Presence**	**1.38**	[1.16;1.55]	**<10**^**−7**^
MIGRATION			
Western path			
**Eastern path**	**1.04**	[0.91;1.24]	**<10**^**−7**^
POPULATION	1.66 10^−7^	[1.66 10^−7^;2.21 10^−7^]	**<10**^**−2**^

Significant variables are highlighted in bold characters.

TMPJUL: Monthly anomalies for July from the perennial mean monthly temperature.

MNDWI21: Modified Normalized Difference Water Index.

λ: Weighted average of the number of infected districts amongst the neighborhood the previous year.

Applying the model to 2012 and 2013 data with the best threshold identified through the analysis of the ROC curve (threshold = 0.014, determined by the observed prevalence method), specificity was 0.639 (95% CI 0.633-0.646) and sensitivity was 0.872 (95% CI 0.841-0.875) for 2012 data and specificity was 0.739 (95% CI 0.729-0.749) and sensitivity was 0.788 (95% CI 0.782-0.805) for 2013 data.

**Figure 4:**
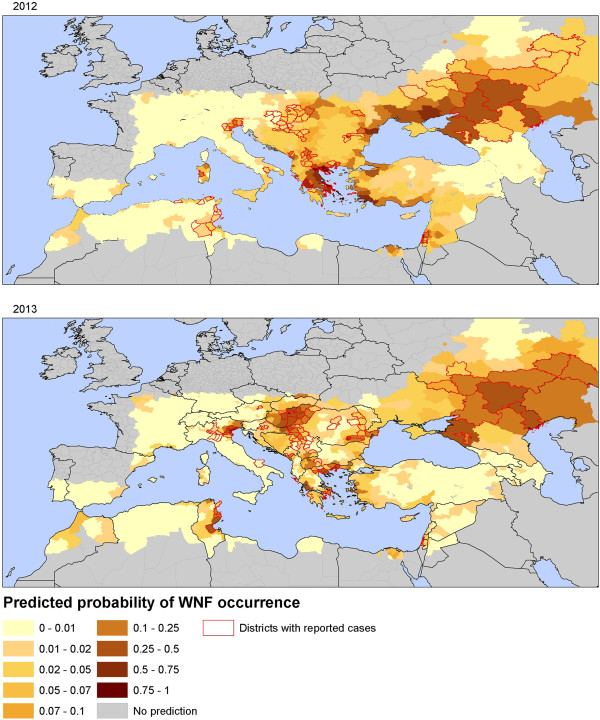
Map of predicted probability of WNV infection based on environmental predictors, Europe and neighboring countries, 2012 and 2013.

Figure 4 shows the probability of WNV infection per district in 2012 and 2013 as predicted by the model (see Additional file 2: Figure S1, for 2002–2011 risk maps). The maps highlight spatial heterogeneity of the risk of WNV occurrence throughout Europe. In 2012 higher risk values were predicted for countries of Central and Eastern Europe, Turkey, Israel, and Tunisia. WND cases were reported in all of the predicted high risk areas, excepted in Ukraine, and Turkey. In 2013, Tunisia, Northern Italy, Northern Greece, Central Europe and South Russia presented the highest predicted values in agreement with main areas of transmission.

WNV is a vector-borne pathogen of global importance since it is the most widely distributed of the encephalitic flaviviruses [14]. Thus, in this study we tested environmental variables as risk factors for WNV transmission in Europe and neighboring countries. Our results identified the anomaly of summer temperatures (July), the anomaly of the MNDWI in early June (June 9^th^-16^th^), the occurrence of a WND outbreak the year before, the human population, the presence of wetlands and the type of passerine flyways as risk factors for WNV transmission in Europe.

Thus, our study based on 2002–2011 WND data confirmed the conclusions of Paz et al. (2013) on the linkage between the 2010 heat and the WND eruptions in Europe and its neighboring countries. Similar associations between high temperatures and WND eruption were found in other locations, especially in studies from North America. In a comprehensive study of 17 states in the United States, positive associations with increasing temperature was detected over each of the four weeks prior to symptom onset [15]. Positive relations with heat were found in additional studies for Georgia [16], California [17], Illinois [18], Connecticut [19], and even northwards in the Canadian Prairies [20].

Reisen et al. (2006) indicated that WNV dispersed into new areas during years with above-normal temperatures and that implication during the following year occurred in summers with above- or normal temperatures. This was the case in Europe, as a comparison between the WND distributions in 2010, 2011 and 2012 (Figure 1, Table 1) and the July temperature anomalies (Figure 2a) suggests that the limited appearance of WND in 2011 was related to less warm summer conditions. When the temperature increased in summer 2012, WND number of cases raised (in 2011, 127 and 212 WND cases were reported in the EU and neighboring countries, respectively; increasing in 2012 to 242 and 693 respectively) [11].

These observations are consistent with our results identifying λ, the weighted average of the number of infected districts in a close neighborhood the year before as risk factor for WNV transmission. They support the hypothesis that WNV has the capacity to persist locally in Europe after a first introduction, probably through survival in overwintering mosquitoes [21,22] or in wild bird populations [23,24]. This epidemiological pattern was only recently observed in Europe, the Italian 2008 outbreak marking a change in the epidemiology of the disease [25].

Our study stressed the importance of water bodies in the risk of WNV transmission, our results showing that areas including wetlands with positive anomalies of the MNDWI in June are more at risk. The role of water bodies in WNV transmission is related to the availability of breeding sites for the mosquito vector populations and may be complex: on one hand, large areas of surfacewater may favor vector abundance and WNV transmission as demonstrated in other studies [26], but in drought conditions, the reduced number of water pools may lead to higher interactions between wild birds and mosquitoes [27]. Our findings support the first hypothesis, suggesting that in Europe, above average surface of water in June, favoring mosquito proliferation before the summer months, is a significant risk factor for WNV transmission. Yet, entomological field observations are needed at the European scale to assess i) the distribution and dynamics of WNV vectors in Europe and ii) the relationship between mosquitoes and MNDWI dynamics, assessing whether an increase of MNDWI, which is related to an increase of water bodies, is a reliable proxy for an increase of available breeding sites of WNV vectors. On the other hand, our study highlights that although water surfaces are generally closely linked to rainfall events, water indices such as the MNDWI might be more relevant as proxies of WND risk, as the association between WND outbreaks occurrence with precipitation was not consistent [28]. The inconsistent linkages between WND eruptions and precipitation amounts appear in the literature for other regions. For instance, Frost et al. (2012) noted that the eruption of acute equine encephalitis (virulent strain of WNV_KUN_) in 2011 in Australia was followed by extensive floods across eastern Australia that created ideal breeding conditions for freshwater *Culex annulirostris*[29]. In studies from the USA, Soverow et al. (2009) found positive associations with rainfall in the months preceding the disease outbreaks; in contrast Shaman et al. (2002) demonstrated that heavy rainfall might lead to a negative association by flushing the ditches and drainage channels used by *Culex* larvae [30]. On the other side, drought can facilitate population outbreaks of some species of mosquitoes. It was shown by DeGroote et al. that prior drought contributed to the initial United States WNV outbreak [31].The predictions of the best model applied to 2012 and 2013 data performed well according to the AUC, sensitivity and specificity indices (Figure 3). Thus, an important part of the variability in WNV transmission in Europe at the district level can be captured by environmental drivers. Yet, other factors related to the presence and abundance of hosts (wild birds) and vectors (mosquito species) of WNV should be studied to better understand the relation between WNV transmission and environmental conditions. To achieve this, entomological and ornithological field studies are required to identify the potential hosts and vectors of WNV in different European locations. Serological surveys on wild avifauna in European and African countries are also needed to confirm and better understand the importance of the type of passerine flyways (Eastern or Western pathway) to predict the risk of WND occurrence in Europe. Expansion of this work should incorporate entomological and ornithological information to improve our understanding of WNV transmission cycles, and model predictions.

Environmental indices derived from satellite remote sensing have been widely used to study environmental risk factors of vector-borne diseases in the last decades [32,33]. Our study also demonstrates that remote sensing medium spatial resolution imagery as MODIS can provide relevant environmental indicators for studies on WNV risk, as it was also recently shown in Northern America [34]. This analysis should include high resolution geolocation of WND cases (i.e. sub-district level) to better address relationship between outbreaks and monitoring of vegetation and water resources.

As in most epidemiological studies, our analysis is dependent on the quality of the original dataset, which suffers from biases related to possible under-detection and under-reporting of WND outbreaks. Indeed, presence prediction can be wrongly interpreted as false positives due to under reporting by national surveillance systems. To limit these biases of possible heterogeneity in space and time of WND surveillance systems, we considered only presence/absence data per district instead of incidence data, and a bootstrap approach was applied to construct confidence intervals. In the future, harmonization of WND surveillance systems across European member states will contribute to improve the epidemiological datasets for analysis.

## Conclusion

This study contributes to a better understanding of the environmental and climatic drivers of WNV transmission in Europe and provides the basis for further integration of environmental information together with WNV surveillance programs in Europe, including surveillance of human cases, serological surveillance of domestic and wild avifauna, and entomological surveillance, as recommended by [13,35]. Our results suggest that risk maps for WNV transmission could be built based on updated anomalies of temperatures and MNDWI. Use of those two environmental risk factors would allow for a better integration of environmental monitoring into public health surveillance systems.

## Methods

### Epidemiological data

**Figure 5:**
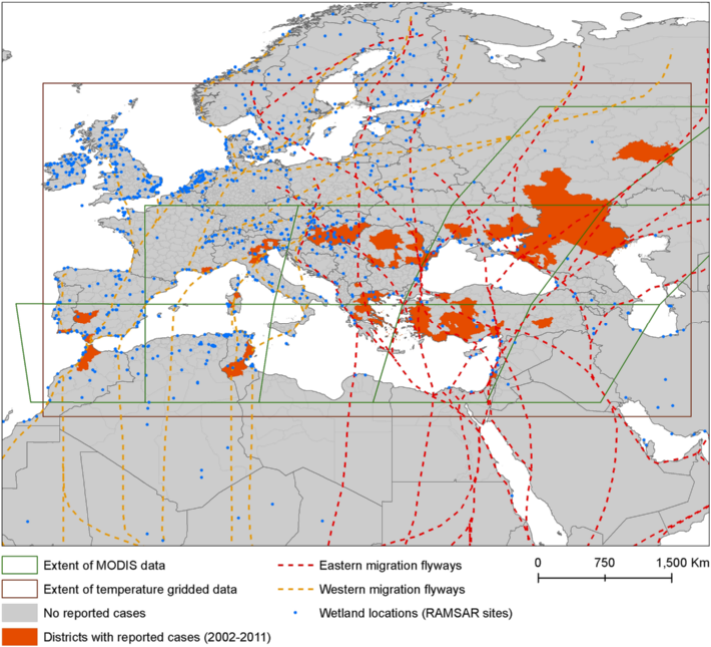
**Study area: location of the West Nile fever outbreaks, Europe and neighbouring countries, 2002–2011, and extent of environmental datasets.** Note: the brown box represents the extent of temperature dataset; the green boxes represent the extent of the MODIS tiles.

Data series of confirmed cases of WND in humans reported in Europe and its neighboring countries between 2002 and 2013 were compiled at the district level to define presence of human case (Table 1, Figure 5). The WND data was assembled through a systematic review of the scientific literature using the MEDLINE database, Embase, and Scopus. Abstracts were screened to identify epidemiological information and the full text articles were retrieved to obtain the precise location of WND cases. In addition, a systematic screening was conducted using the Global Infectious Diseases and Epidemiology Online Network, Promed for central Europe and meeting reports and newsletters of scientific projects on WND of the Framework Programme for Research and Technological Development funded by European union [36].

The screening described above was merged with West Nile human cases on West Nile fever surveillance conducted by ECDC during the transmission season in Europe [11].

### Population data

Within European Union, the nomenclature of territorial units for statistics classification (NUTS) was used for administrative unit at level 3 with population estimate of 2010 [37]. For outside of the European Union, administrative units data were provided by the Global Administrative Unit Layers (GAUL) project and population estimates for the year 2010 were derived from the Gridded Population of the World (GPW) dataset [38].

### Ecological and climatic data birds’ migration routes

Passerine fly ways were digitized in order to categorize administrative units in two categories of migration flyway (western and eastern) according to the migration flyways of Western Palearctic Passerines South Eastern European bird migration network [39].

### Wetlands

The presence or absence of wetlands in a district was defined according to Ramsar Sites Information Service form the Convention on Wetlands of International Importance [40]. Quality control was made by image interpretation using Google Earth and the Global Lakes and Wetlands Database (GLWD, level data 1 and 2) maintained by the World Wide Fund for Nature and the Center for Environmental Systems Research, University of Kassel, Germany [41].

### Time-dependent variables

We examined three time-dependent environmental variables as predictors of WNV risk: the temperature, an index related to the state of the vegetation, the NDVI, and an index related to the state of the water bodies, the MNDWI. The two latter were derived from MODIS imagery. These three indices vary seasonally and inter-annually, and are hypothesized to influence mosquito population dynamics, and WNV transmission [31,34,42].

Temperature has been found to be associated with WND outbreaks in the Old [12] and New World [34]. Indeed, elevated temperatures favor vector competence, accelerate mosquito development and reproduction rates, thus influencing mosquito-to-vertebrate transmission rates [43,44]. The gridded data of monthly mean of air temperature for the region between 30°N-60°N and 10°W-55°E was obtained from the NOAA NCEP-NCAR database [45,46] for each month from January 1981 to December 2013, (Table 3, Figure 5).

**Table 3:** Characteristics of the environmental datasets

	**Spatial resolution**	**Time frequency**	**Time-period**
Temperature	1.875° (~150 km)	1-month	1981-2013
MODIS NDVI and MNDWI indices	500 m	8-day	2002-2013

On the other hand, vegetation indices such as the NDVI have been identified as risk factors for WND outbreaks occurrence in previous North American studies [34,47]. NDVI may serve as an indicator of environmental conditions suitable for vegetation growth and emergence of mosquito populations [34].

The presence of water bodies was identified as another environmental WND risk factor [26], because large standing water resources may lead to an upsurge of mosquito populations. Several methods are used in radar and optical remote sensing to delineate water bodies and map flooded areas. The Modified Normalized Difference Water Index (MNDWI) is particularly suited to the detection of free water [48].

To derive NDVI and MNDWI values, MODIS data products were acquired from Land Process Distributed Active Archive Center (LP DAAC). MODIS Terra 8-day composite images of surface reflectance estimates at 500 m spatial resolution (product MOD09A1) were acquired for all WNV infected countries for a twelve years period (2002–2013) (Table 3, Figure 5). Preprocessing steps consisted in masking the low quality pixels and the pixels covered by clouds using the surface reflectance quality file, and performing a linear temporal interpolation of the masked pixels using the Time Series Generator (TiSeG) freeware [49].

NDVI and MNDWI temporal series were computed from the reflectance values of the cleaned images according to Equations 1 and 2:

(1) $$\mathrm{NDVI}=\left(\mathrm{NIR}-R\right)/\left(\mathrm{NIR}+R\right)$$

(2) $$\mathrm{MNDWI}=\left(G-\mathrm{SWIR}\right)/\left(G+\mathrm{SWIR}\right)$$

with NIR: reflectance in near infrared range, R: reflectance in red wavelength; G: reflectance in green wavelength; SWIR: reflectance in short wave infrared range.

Image processing was performed using ENVI IDL software 4.8 (Exelis, Boulder, C0, USA).

### Quantifying anomalies of temperature, vegetation and water indices relative to the long term average

First, the long-term average and standard deviation of each of the environmental indices were computed on monthly bases for the temperature data, and on 8-days interval bases for the MODIS NDVI and MNDWI data. The long-term statistics were computed on a 20 years period (1981–2010) and on a 10 years period (2002–2011) for the temperatures and for the MODIS-derived indices, respectively.

Second, the anomaly (*z*) of temperatures, NDVI and MNDWI was calculated for each date *i* (month or 8-days period) as a function of the annual indices *x*_*i*_ and their long-term average and standard deviation values (Equation 3). Study period was selected to begin in March (before WND outbreaks) and end in August.

(3) $$z_{i}=\frac{x_{i}-{\bar{x}}_{i}}{\sigma_{i}}$$

Finally, the mean anomalies of temperatures, NDVI and MNDWI were computed for each district and each month and MODIS 8-days period.

Analyses were performed using ArcGIS 10.1 and Spatial analyst extension (ESRI, Redlands, CA).

### Statistical analysis

The status of infection by WNV in relationship to ecological and climatic variables was analyzed at the district level (n = 1113 spatial units). For each year, each district was categorized as ‘infected’ if WND human cases were reported there that year, and as ‘non infected’ otherwise. We used the data from 2002 to 2011 to fit the models, and the data for 2012 and 2013 for external validation.

The probability of a district to be infected by WNV was assessed using logistic regression models, with the status of infection as the response variable, and as explanatory variables the population, the presence of wetlands, the presence of birds’ migratory routes, the anomalies of temperature, NDVI and MNDWI. We also tested as explanatory variable the occurrence of a WND outbreak the previous year, considering that WNV could persist locally through survival in overwintering mosquitoes [25]: for each year *i* and each district *j*, a synthetic index (λ) of WND outbreak occurrence in the neighborhood of district *j* was defined as the weighted average of the number of infected districts amongst a set of *n*_*j*_ neighbours of district *j* according to Equation 4:

(4) $$\lambda_{i,j}=\frac{\sum_{k=1}^{n_{j}}y_{i-1,k}.w_{\mathrm{jk}}}{n_{j}+1}$$

with *yi,j*: infected status of district *j*, year *i* (0: ‘non infected’; 1: ‘infected’); *n*_*j*_: number of neighbouring districts of district j; the weight *w*_*jk*_ given to district k is 1 if k ≠ j and 2 otherwise.

Univariate analysis. Explanatory variables were tested one at a time to test associations with district WNV status. Significant variables in this preliminary univariate screening analysis at 0.05 p-value were kept for analysis of co-linearity.

Multivariate analysis. Multivariate logistic models were built to examine the role of explanatory variables, having adjusted for other variables. All possible models including variables significant in the univariate analysis were fitted. In case of co-linearity, the variable corresponding to the earlier date was kept. We used backward model selection based on AIC to select the best model based on both model fit and model parsimony [50]. A bootstrap procedure (1,000 replicates) was applied to estimate the 95% confidence interval (95% CI) of the logistic regression models’ coefficients, selecting randomly each time from the original set of 1113 districts 90% of infected districts between 2002 and 2011 (n = 98) and 90% of non-infected districts (n = 903).

Validation. The predictive accuracy of the final model was assessed using the ROC (Receiver Operating Characteristic) curve [51]. The area under curve (AUC) of the ROC curve, sensitivity and specificity indices were computed using data from 2002 to 2011 (internal validation) and data from 2012 and 2013 (external validation). The greater the AUC, the closer the predictions are to the observed data. 95% CI were estimated using the same bootstrap procedure described above (1,000 replicates).

Generation of predictive maps. The final model was used to predict the probability of WNV infection for the entire Europe and neighboring countries by applying the model to observed data from all districts of the studied area from 2002 to 2013.

Analyses were performed using the ‘R’ freeware and additional ‘spdep’, ‘lme4’, and ‘PresenceAbsence’ packages [52].

## Abbreviations

AIC: Akaike information criterion; AUC: Area under curve; CI: Confidence interval; GAUL: Global administrative unit layers; GPW: Gridded population of the world; GLWD: Google earth and the global lakes and wetlands database; NDVI: Normalized difference vegetation index; NUTS: Nomenclature of territorial units for statistics classification; MODIS: Moderate resolution imaging spectroradiometer; MNDWI: Modified normalized difference water index; ROC curve: Receiver operator characteristic curve; WND: West nile disease; WNND: West nile neuro invasive disease; WNF: West nile fever; WNV: West nile virus.

## Competing interests

Competing financial interests declaration: none declared.

## Authors’ contributions

JCS initiated the project entitled: Mosquito-Borne Diseases Determinants (OJ/2012/02/16 – PROC/2012/015) and developed the strategy for a NWF analysis. BS compiled the epidemiologic data and AT led the analysis with contributions from BS, SP, MR, AD, VC and JCS. All authors read and approved the final manuscript.

## Supplementary Material

Additional file 1: Table S1 Results of univariate analysis: significant variables in univariate screening analysis at 0.05 p-value. Click here for file

Additional file 2: Figure S1 Maps of predicted probability of WNV infection based on environmental predictors, and West Nile fever outbreaks, Europe and neighbouring countries, 2002–2013. Click here for file
