# Supplementary material for: Environmental predictors of West Nile fever risk in Europe
Source: Int J Health Geogr. 2014 Jul 1;13:26. doi: 10.1186/1476-072X-13-26 (PMC4118316; doi:10.1186/1476-072X-13-26)
Supplement: Additional file 1: Table S1 — Results of univariate analysis: significant variables in univariate screening analysis at 0.05 p-value. [file 1476-072X-13-26-S1.doc]

| **Covariate** | **Estimate** | **p-value** |
| --- | --- | --- |
|  | 5.64 | <0.0001 |
| TMPAUG | 0.45 | <0.0001 |
| WETLAND | 1.58 | <0.0001 |
| MNDWI21 | 0.88 | <0.0001 |
| POP | 2.45 10^−7^ | <0.0001 |
| MIGRAT | 1.17 | <0.0001 |
| TMPJUL | 0.33 | <0.0001 |
| MNDWI20 | 0.56 | <0.0001 |
| MNDWI22 | 0.50 | 0.0001 |
| MNDWI15 | 0.45 | 0.0006 |
| MNDWI26 | 0.35 | 0.0067 |
| NDVI21 | −0.07 | 0.0168 |
| MNDWI18 | 0.30 | 0.0173 |
| NDVI20 | −0.07 | 0.0168 |
| NDVI30 | −0.07 | 0.0294 |
| NDVI22 | −0.06 | 0.0331 |
| MNDWI10 | 0.23 | 0.0472 |
