# Supplementary figures and images for: Environmental predictors of West Nile fever risk in Europe
Source: Int J Health Geogr. 2014 Jul 1;13:26. doi: 10.1186/1476-072X-13-26 (PMC4118316; doi:10.1186/1476-072X-13-26)

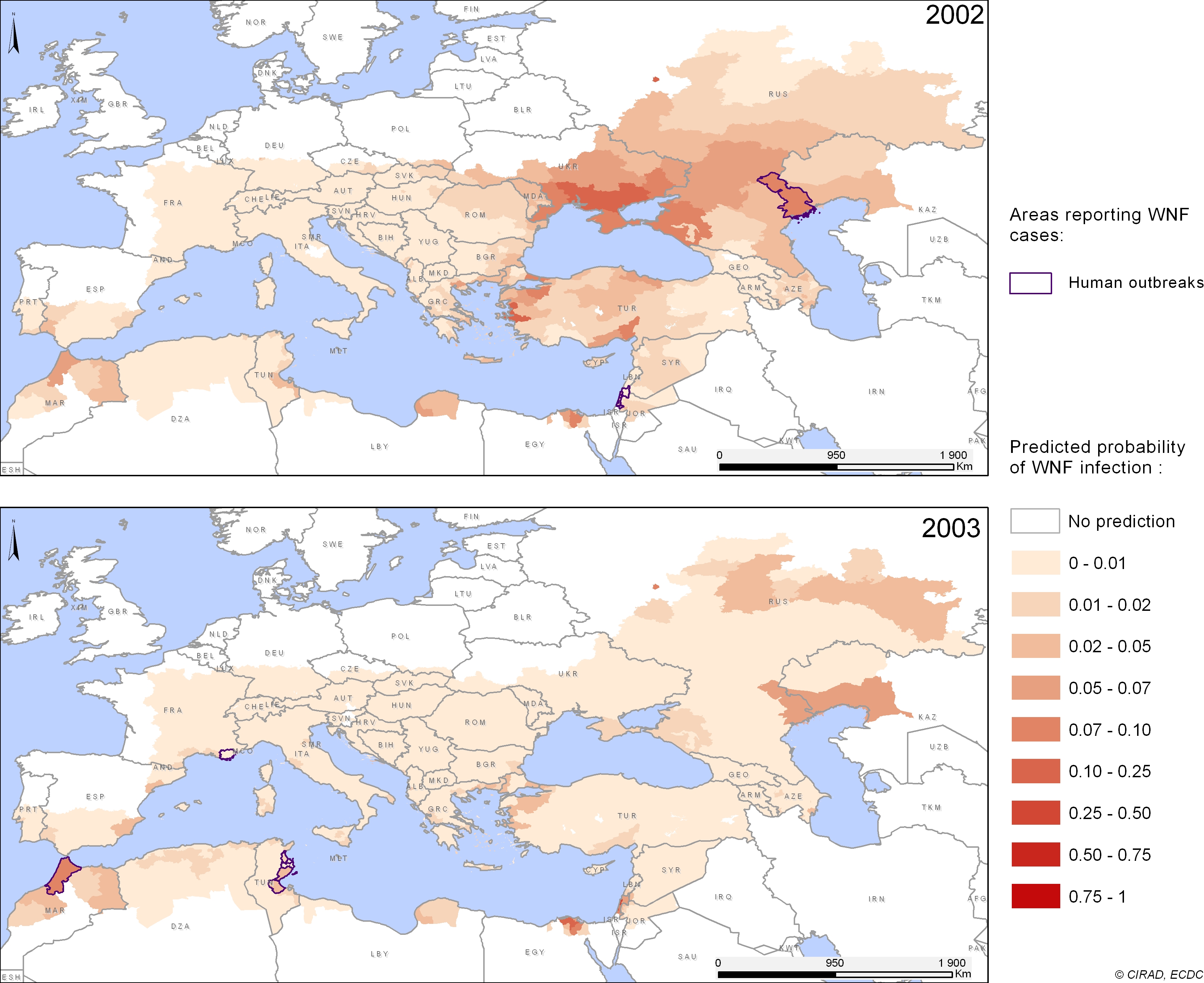

Supplement: Additional file 2: Figure S1 — Maps of predicted probability of WNV infection based on environmental predictors, and West Nile fever outbreaks, Europe and neighbouring countries, 2002–2013. [file 1476-072X-13-26-S2.zip › 1104470634121792_add1.jpeg]

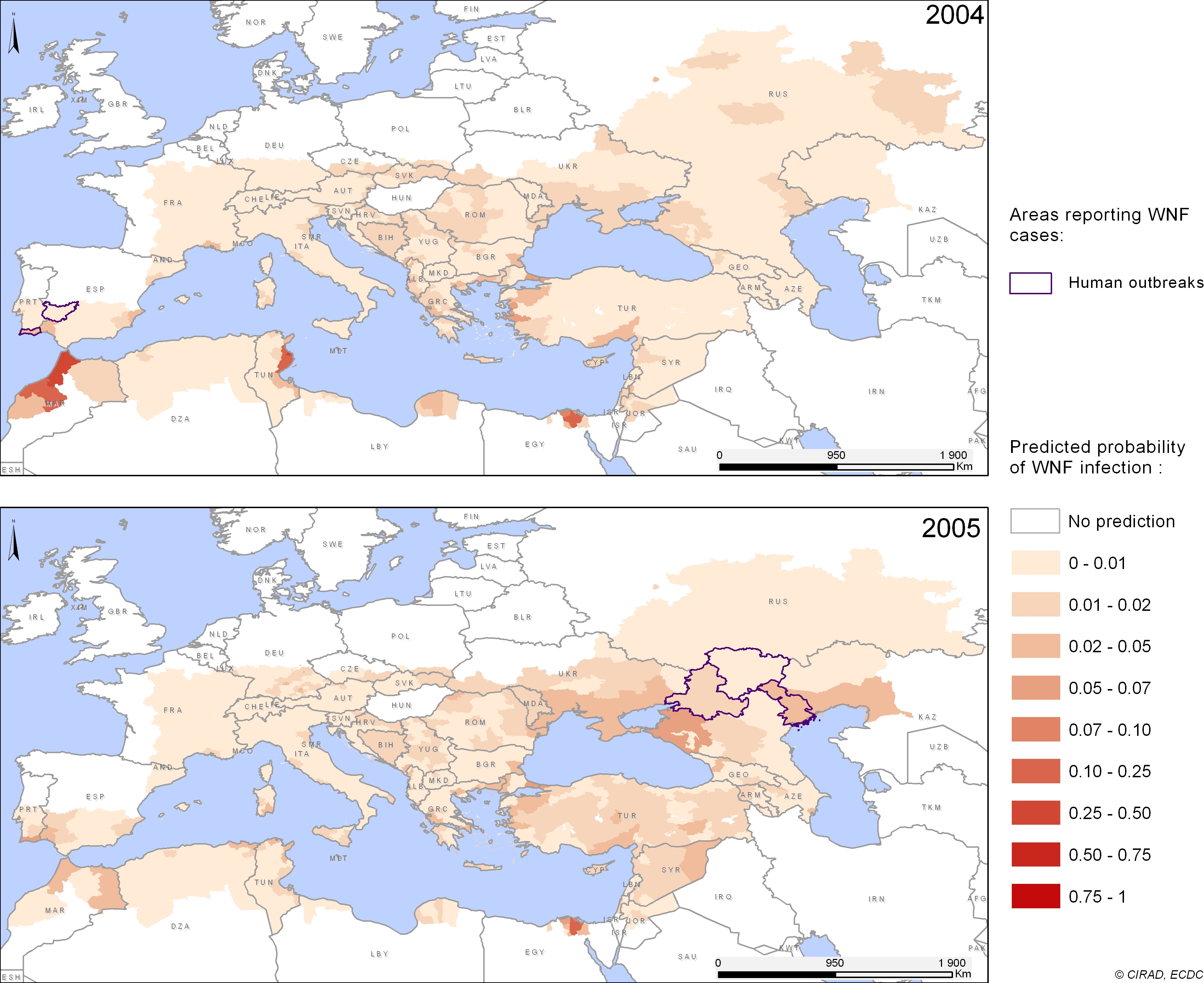

Supplement: Additional file 2: Figure S1 — Maps of predicted probability of WNV infection based on environmental predictors, and West Nile fever outbreaks, Europe and neighbouring countries, 2002–2013. [file 1476-072X-13-26-S2.zip › 1104470634121792_add2.jpeg]

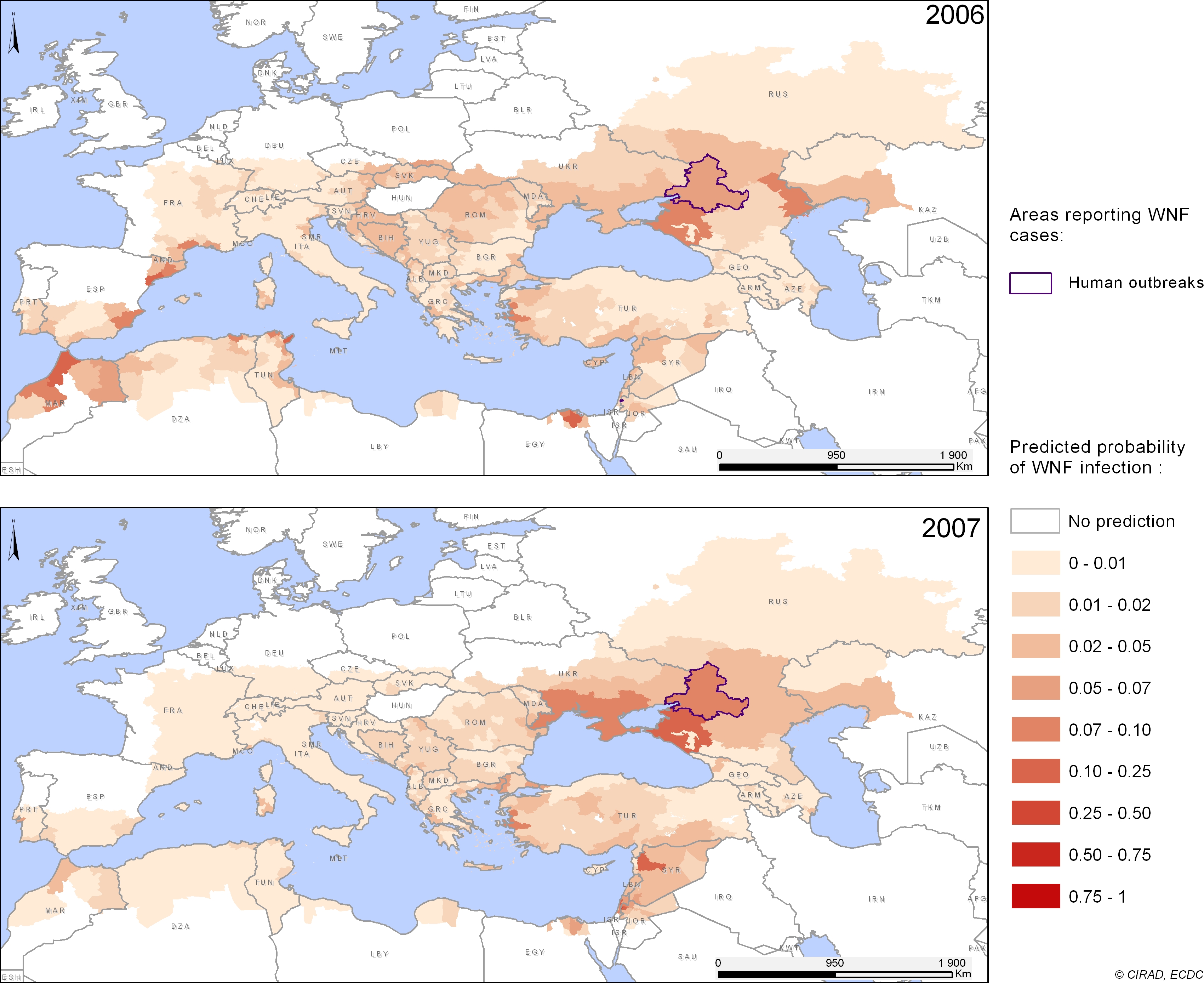

Supplement: Additional file 2: Figure S1 — Maps of predicted probability of WNV infection based on environmental predictors, and West Nile fever outbreaks, Europe and neighbouring countries, 2002–2013. [file 1476-072X-13-26-S2.zip › 1104470634121792_add3.jpeg]

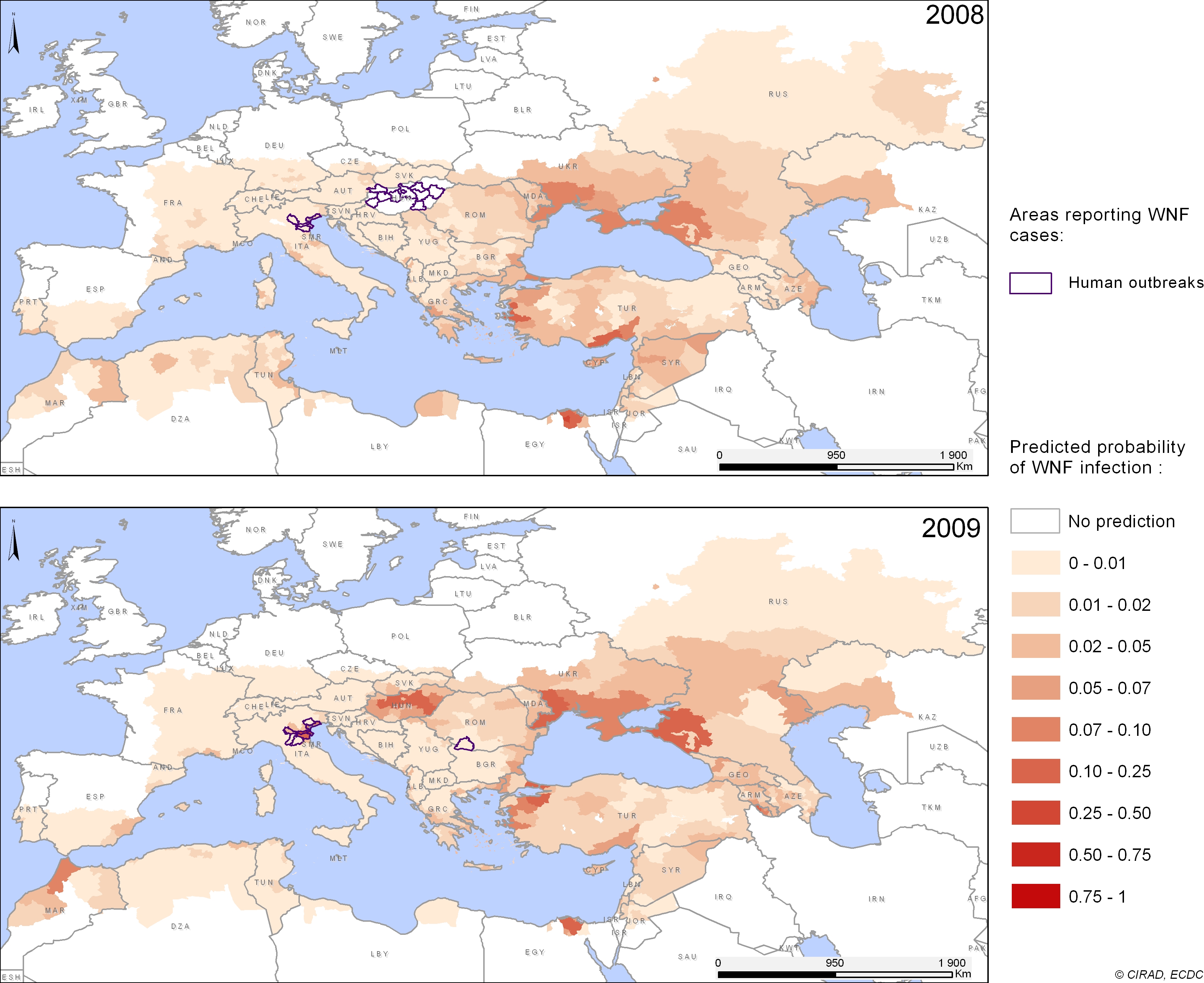

Supplement: Additional file 2: Figure S1 — Maps of predicted probability of WNV infection based on environmental predictors, and West Nile fever outbreaks, Europe and neighbouring countries, 2002–2013. [file 1476-072X-13-26-S2.zip › 1104470634121792_add4.jpeg]

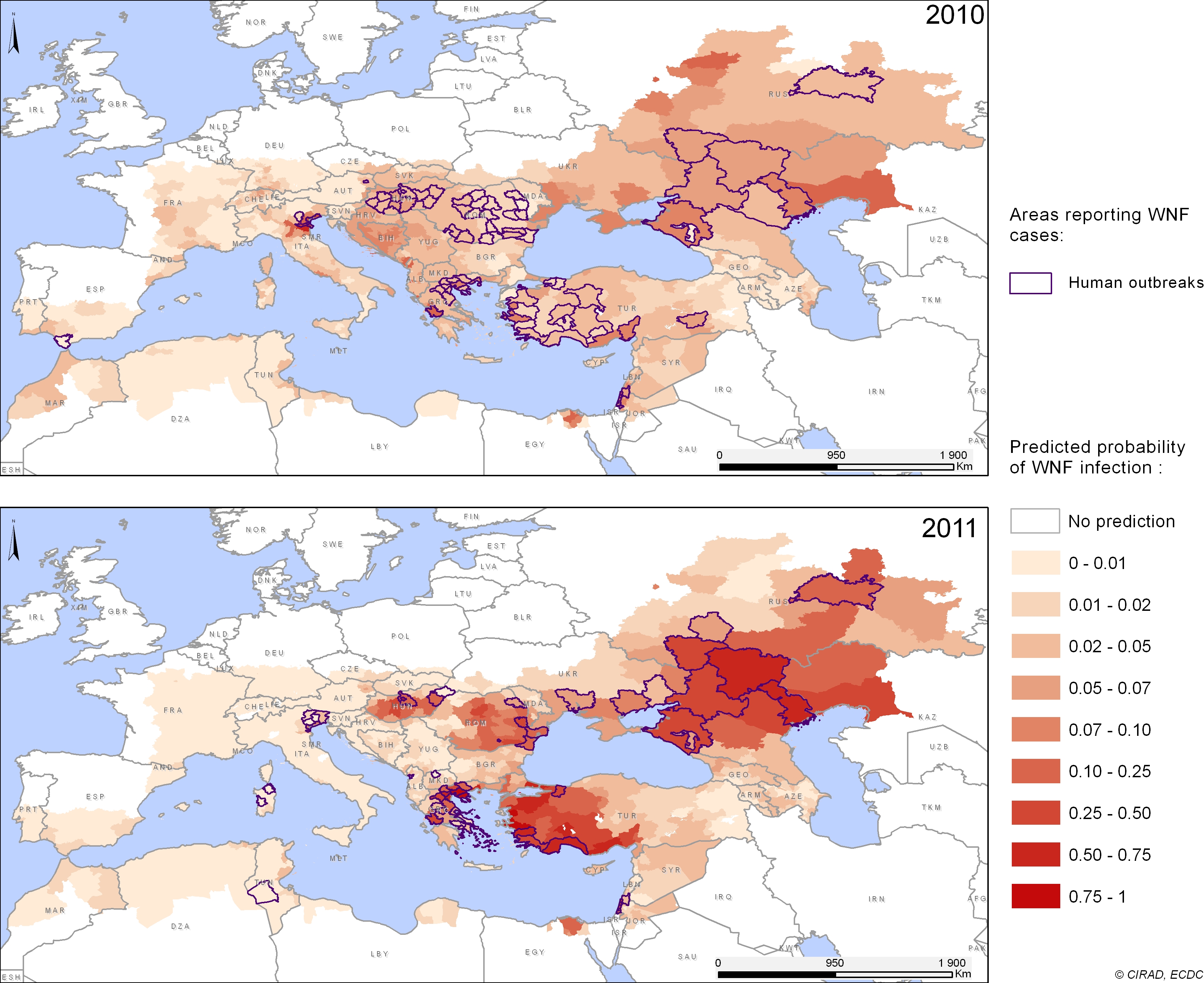

Supplement: Additional file 2: Figure S1 — Maps of predicted probability of WNV infection based on environmental predictors, and West Nile fever outbreaks, Europe and neighbouring countries, 2002–2013. [file 1476-072X-13-26-S2.zip › 1104470634121792_add5.jpeg]

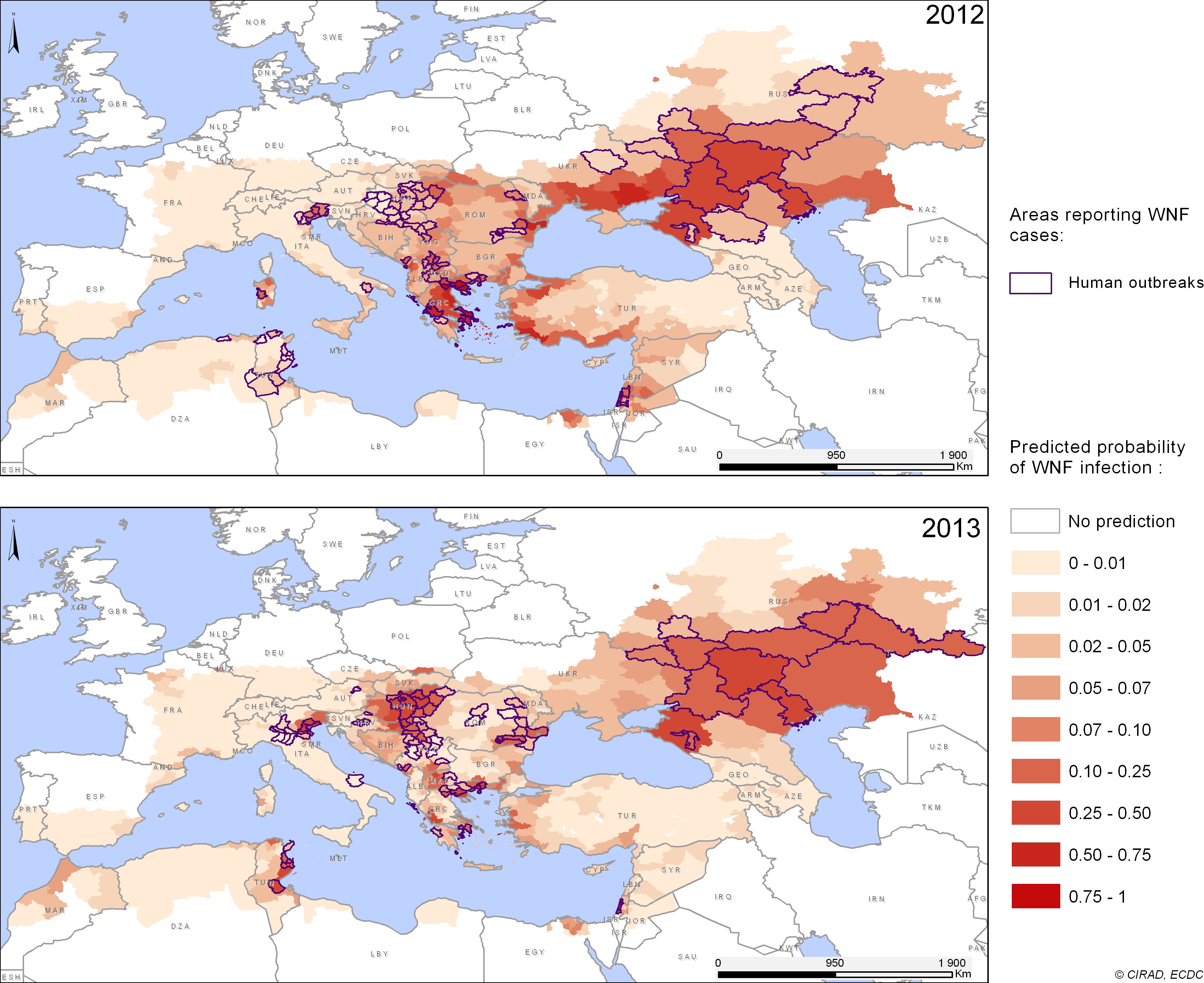

Supplement: Additional file 2: Figure S1 — Maps of predicted probability of WNV infection based on environmental predictors, and West Nile fever outbreaks, Europe and neighbouring countries, 2002–2013. [file 1476-072X-13-26-S2.zip › 1104470634121792_add6.jpeg]
